# 7-Methoxytacrine-*p*-Anisidine Hybrids as Novel Dual Binding Site Acetylcholinesterase Inhibitors for Alzheimer’s Disease Treatment

**DOI:** 10.3390/molecules201219836

**Published:** 2015-12-10

**Authors:** Jan Korabecny, Martin Andrs, Eugenie Nepovimova, Rafael Dolezal, Katerina Babkova, Anna Horova, David Malinak, Eva Mezeiova, Lukas Gorecki, Vendula Sepsova, Martina Hrabinova, Ondrej Soukup, Daniel Jun, Kamil Kuca

**Affiliations:** 1 Biomedical Research Centre, University Hospital Hradec Kralove, Sokolska 581, 500 05 Hradec Kralove, Czech Republic; jan.korabecny@fnhk.cz (J.K.); martin.andrs@unob.cz (M.A.); Eugenie.nepovimova@unob.cz (E.N.); Rafael.dolezal@fnhk.cz (R.D.); katerina.babkova@unob.cz (K.B.); anna.horova@unob.cz (A.H.); david.malinak@gmail.com (D.M.); eva.mezeiova@gmail.com (E.M.); lukasgorecki@seznam.cz (L.G.); vendula.sepsova@unob.cz (V.S.); martina.hrabinova@unob.cz (M.H.); ondrej.soukup@fnhk.cz (O.S.); daniel.jun@unob.cz (D.J.); 2 Department of Toxicology and Military Pharmacy, Faculty of Military Health Sciences, Trebesska 1575, 500 01 Hradec Kralove, Czech Republic; 3 National Institute of Mental Health, Topolova 748, 250 67 Klecany, Czech Republic

**Keywords:** Alzheimer’s disease, acetylcholinesterase, butyrylcholinesterase, tacrine, 7-methoxy-tacrine, MTDLs

## Abstract

Alzheimer’s disease (AD) is a debilitating progressive neurodegenerative disorder that ultimately leads to the patient’s death. Despite the fact that novel pharmacological approaches endeavoring to block the neurodegenerative process are still emerging, none of them have reached use in clinical practice yet. Thus, palliative treatment represented by acetylcholinesterase inhibitors (AChEIs) and memantine are still the only therapeutics used. Following the multi-target directed ligands (MTDLs) strategy, herein we describe the synthesis, biological evaluation and docking studies for novel 7-methoxytacrine-*p*-anisidine hybrids designed to purposely target both cholinesterases and the amyloid cascade. Indeed, the novel derivatives proved to be effective non-specific cholinesterase inhibitors showing non-competitive AChE inhibition patterns. This compounds’ behavior was confirmed in the subsequent molecular modeling studies.

## 1. Introduction

**Figure 1:**
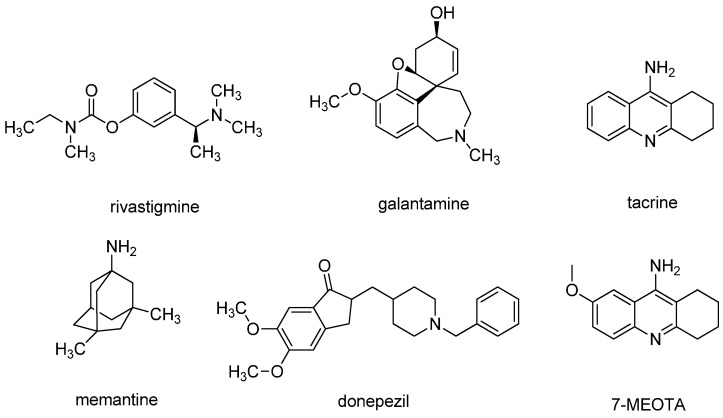
Chemical structures of AChEIs and NMDAR antagonist memantine for the AD treatment.

Alzheimer’s disease (AD) is a progressive and fatal neurodegenerative disorder characterized by memory loss and personality changes. AD is also considered as one of the biggest global public burden, currently affecting more than 44 million people worldwide, a number estimated to increase up to 150 million people by 2050 [1,2]. Although many factors have been implicated in AD, its etiology is not completely clear. Finding the solutions for AD in terms of suitable therapy has been a greater challenge and for the past few decades many researchers and pharmaceutical companies have been optimistically working towards this goal. Diverse pathological factors have been showed to be responsible for AD pathology. Among them, extracellular deposits of β-amyloid (Aβ), hyper-phosphorylated neurofibrillary tangles (NFT) of tau protein, reactive oxygen species (ROS), metal imbalance and disrupted cholinergic system have received particular attention [3,4,5,6,7]. The latter pathological feature, being the main postulate of the so called cholinergic hypothesis, is well established. Indeed, postmortem brains have confirmed low levels of cholinergic markers [8]. Two types of cholinesterase (ChE) enzymes have been found in the central nervous system, including acetylcholinesterase (AChE; E.C. 3.1.1.7) and butyrylcholinesterase (BChE; E.C. 3.1.1.8), both being responsible for the termination of synaptic cholinergic transmission by rapid hydrolysis of acetylcholine (ACh). Despite the impressive amount of progress in understanding the molecular mechanisms behind AD, ChE inhibitors such as tacrine, donepezil, rivastigmine and galantamine represent currently almost the only employed approach for the treatment of AD (Figure 1) [9]. Apart from ChE inhibitors, the *N*-methyl-d-aspartate receptor (NMDAR) antagonist memantine has proved to be an efficacious treatment for patients in later stages of AD (Figure 1) [10].

Tacrine was the first drug approved by the FDA for AD treatment in 1993. Tacrine demonstrated an ability to cross the blood-brain barrier (BBB) quite easily and to inhibit central AChE in the sub-micromolar range [11]. The toxicity of tacrine is a consequence of the formation of several hydroxylated derivatives during its liver metabolization by the microsomal cytochrome P450 enzyme family [12]. This, together with its gastrointestinal side effects, difficulty in dosing regimen and required periodic blood monitoring, resulted in tacrine being withdrawn from the pharmaceutical market. In a search for the less toxic ChE inhibitors with preserved pharmacological profile, 7-methoxytacrine (7-MEOTA) showed better toxicological profile than tacrine (Figure 1) [13,14,15].

**Figure 2:**
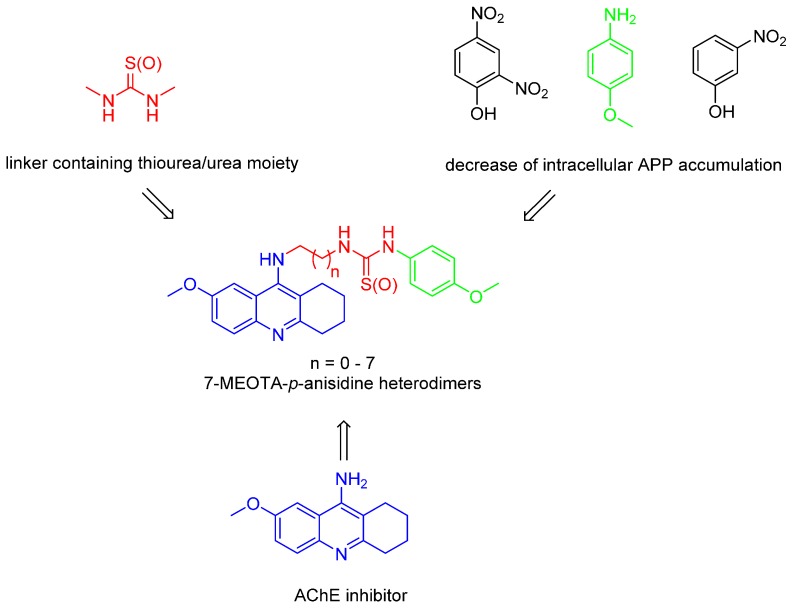
Design strategy for novel 7-MEOTA-*p*-anisidine hybrids.

In a continuation of our research [16,17,18,19,20,21], herein we combined a less toxic tacrine derivative, namely 7-MEOTA, with *p*-anisidine connected through an alkyl tether containing thiourea or urea moieties. The results of previous studies have shown that both tacrine and 7-MEOTA are capable of binding to the peripheral anionic site (PAS) as well as to the catalytic anionic site (CAS) of AChE, depending on the structural features of the second attached moiety [22]. The length of the alkyl chain plays an important role in providing proper contact to both crucial parts of the enzyme as shown previously in many studies [23,24]. This might be different for AChE and BChE due to their conformational diversity [25]. We [26,27] and others [28,29] have shown that introduction of thiourea and/or urea groups into the linker might be beneficial in terms of increasing the inhibitory activity against AChE/BChE. Finally, the synthetic feasibility led us to combine 7‑MEOTA with *p*-anisidine, a commercially available chemical compound with a potential to decrease intracellular accumulation of amyloid precursor protein (APP), the precursor of neurotoxic Aβ found in the brains of AD patients (Figure 2) [30]. Novel compounds presented in this study may help to move forward in neurological disorders like AD.

## 2. Results and Discussion

### 2.1. Chemistry

**Scheme 1:**
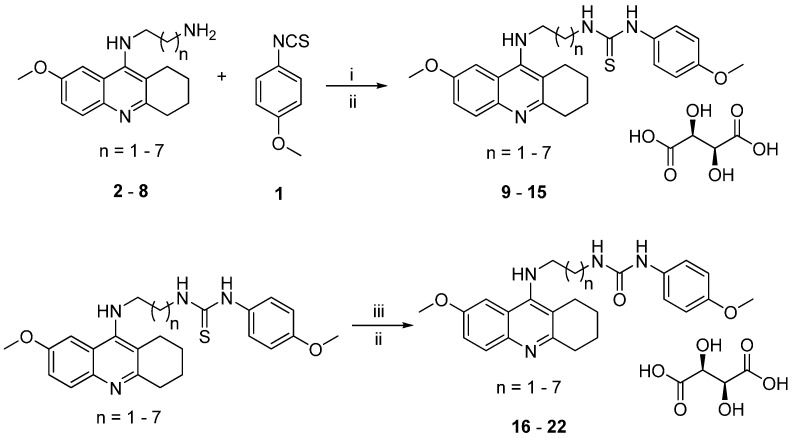
Synthesis of novel 7-MEOTA-*p*-anisidine heterodimers **9**–**22**. *Reagents and conditions*: (i) CHCl_3_, 24 h, r.t.; (ii) l-(+)-tartaric acid, EtOH, 24 h, r.t.; (iii) 2,4,6-trimethylbenzonitrile-*N*-oxide, dichloromethane, 24 h, r.t.

The synthesis of the target 7-MEOTA-*p*-anisidine heterodimers was carried out according to the procedure depicted in Scheme 1. Firstly, *p*-anisidine was quantitatively converted to 1-isothio-cyanato-4-methoxybenzene (**1**) with carbon disulfide (CS_2_) using di-*tert*-butyl dicarbonate (Boc_2_O), triethylamine (TEA) and catalytic amount of 4-(dimethylamino)pyridine (DMAP) [31]. The second moiety, *N*^1^-(7-methoxy-1,2,3,4-tetrahydroacridin-9-yl)alkane-1, ω-diamines **2**–**8**, were synthesized by following the known procedure [26,27]. The intermediates **2**–**8** were then treated with **1** in chloroform and stirred at room temperature for 24 h to obtain the expected 7-MEOTA-*p*-anisidine thiourea series. These were consequently converted to the corresponding salts **9**–**15** in overall yields of 15%–42% by reaction with l-(+)-tartaric acid under room temperature conditions. For the synthesis of the second target 7-MEOTA-*p*-anisidine urea family (compounds **16**–**22**), we utilized **9**–**15** in the form of free bases which were treated with 2,4,6-trimethylbenzonitril-*N*-oxide. Subsequent conversion of the free urea bases to tartaric salts afforded the title compounds **16**–**22**. All new 7-MEOTA-*p*-anisidine hybrids (**9**–**22**; yields 13%–46%) showed analytical and spectroscopic data in good agreement with their structures (see Experimental Section).

### 2.2. Biological Evaluation of AChE/BChE Activity

In order to investigate the biological profile of novel 7-MEOTA-*p*-anisidine heterodimers **9**–**22**, we used human AChE (*h*AChE) and human BChE (*h*BChE) for the determination of their inhibitory potency following a slightly modified Ellman *et al.* protocol [32,33]. The obtained data were compared to tacrine and 7-MEOTA, used as reference compounds.

As listed in Table 1, all the newly synthesized compounds turned out to be potent inhibitors of both cholinesterases. The IC_50_ were in the moderate to low micromolar range for at least one enzyme. Regarding AChE inhibitory activity, all of the 7-MEOTA-*p*-anisidine hybrids were less potent than tacrine, however, in several cases (compounds **10**, **12**, **14**, **15**, **19**–**22**) they were slightly more active than the parent 7-MEOTA. Compounds containing a thiourea moiety in the linker with longer methylene tethers (**14**, **15**) exerted higher AChE inhibitory activities than shorter ones. 

**Table 1:** Inhibitory activities of newly developed 7-MEOTA-*p*-anisidine hybrids **9**–**22** and reference compounds (tacrine and 7-MEOTA) for *h*AChE and *h*BChE expressed as IC_50_ values.

Compound	*n*	*h*AChE IC_50_ ± SEM (μM) ^a^	*h*BChE IC_50_ ± SEM (μM) ^a^	Selectivity for *h*AChE ^b^
**9**	1	43.6 ± 2.1	1.03 ± 0.1	0.02
**10**	2	6.36 ± 0.5	8.73 ± 0.1	1.37
**11**	3	32.8 ± 9.9	6.04 ± 0.1	0.18
**12**	4	4.9 ± 0.3	13.5 ± 0.7	2.71
**13**	5	10.3 ± 1.3	9.35 ± 0.1	0.90
**14**	6	3.96 ± 0.1	3.13 ± 0.3	0.79
**15**	7	1.36 ± 0.4	10.2 ± 4.7	7.53
**16**	1	44.9 ± 1.4	11.9 ± 2.3	0.27
**17**	2	26.9 ± 5.9	15.9 ± 4.1	0.59
**18**	3	13.8 ± 3.9	9.34 ± 0.1	0.68
**19**	4	1.35 ± 0.3	10.9 ± 1.6	8.07
**20**	5	4.56 ± 0.9	5.75 ± 0.4	1.26
**21**	6	1.72 ± 0.3	1.69 ± 0.2	0.98
**22**	7	2.14 ± 0.6	1.34 ± 0.2	0.63
Tacrine	-	0.32 ± 0.01	0.08 ± 0.001	0.68
7-MEOTA	-	10.0 ± 0.9	17.6 ± 0.8	1.76

^a^ results are expressed as the mean of at least three experiments; ^b^ selectivity for *h*AChE is determined as ratio *h*BChE IC_50_/*h*AChE IC_50_.

Bioisosteric replacement (S → O) into urea-containing counterparts (compounds **16**–**22**) displayed a similar trend in *h*AChE inhibitory activity, with affinity enhancement up to five-eight methylene spacers (compounds **19**–**22**). The most profound inhibitory effect in terms of *h*AChE activity and selectivity towards this enzyme was observed in compound **19** (*h*AChE IC_50_ = 1.35 μM) bearing a urea group with a five methylene linker between both structural motifs. These data are fully consistent with those previously reported for 7‑MEOTA‑adamantylamine hybrids as conjugates containing either thiourea or urea moieties in the linker where five-carbon chain resulted in the most effective AChE inhibitor [26,27]. On the other hand, the most active derivative in the thiourea family **15** (*h*AChE IC_50_ = 1.36 μM) linking 7-MEOTA and *p‑*anisidine by an eight methylene spacer revealed a pattern of inhibition in the same range as the most promising hybrid from the urea family. Such a discrepancy in the linker length between these two conjugates in relationship to AChE inhibition activity might be explained by different orientation in the enzyme active site provided by various interactions (readers are referred to the molecular modeling study results). Compared to tacrine, compounds **15** and **19** appeared to be 4.2-fold weaker inhibitors of *h*AChE.

The biochemical properties of BChE in the course of neurodegenerative diseases also deserve a brief note. Under physiological conditions, a large population of neurons release high levels of ACh and AChE. The severe loss of these neurons during AD leads to ACh and AChE depletion. Moreover, this phenomena is associated with increasing levels of BChE which may therefore overtake the role of AChE in the neurotransmitter hydrolysis in the later stages of the disease [34,35]. Accordingly, particular attention has been also turned to determining the *h*BChE inhibition ability of the novel 7-MEOTA-*p*-anisidine analogues. The inhibitory potency of novel derivatives **9**–**22** towards *h*BChE lies in the micromolar to low-micromolar range, not exceeding the activity of tacrine, however, being 1.1–17.0 fold more potent than parent 7-MEOTA. Moreover, a structure-activity relationship (SAR) for *h*BChE inhibition activity can be drawn. In the thiourea subset (compounds **9**–**15**), the increasing length of the linker affected inhibition properties detrimentally, highlighting the shortest analogue **9** (*h*BChE IC_50_ = 1.03 μM) as the most active. No significant differences in overall *h*BChE affinity were obtained for the urea family. However, 7-MEOTA-*p*-anisidine ureas revealed opposite trends associated with the tether length, where the most active derivative found was the longest one (**22**; *h*BChE IC_50_ = 1.34 μM). Interestingly, our data are inconsistent with the 7-MEOTA-adamantylamine conjugates suggesting that the optimal spacer length for *h*BChE inhibitory ability either for thiourea or urea series ranged between five to seven methylenes [26,27]. Related to tacrine, the most active *h*BChE inhibitors **9** and **22** proved to be 12.9-fold and 16.8-fold weaker inhibitors, respectively.

In summary, derivative **9** was highlighted as the strongest *h*BChE inhibitors in the tested series with the highest selectivity profile towards this enzyme. On the contrary, urea moiety and five carbon linker conferred on the derivative **19** the highest preference for *h*AChE. 

### 2.3. Kinetic Analysis

**Figure 3:**
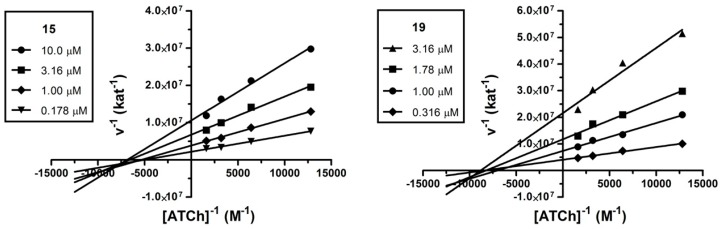
Steady-state inhibition of AChE hydrolysis of acetylthiocholine (ATCh) by compounds **15** and **19**. Lineweaver-Burk reciprocal plots of initial velocity and different substrate concentrations (0.781–6.25 mM) are presented. Lines were derived from a weighted least-squares analysis of data.

The mechanism involved in the AChE inhibition was investigated for the two most potent cholinesterase inhibitors **15** (IC_50_ = 1.36 ± 0.4 μM) and **19** (IC_50_ = 1.35 ± 0.3 μM). We used a kinetic assay in order to obtain information about the mode of inhibition and binding site of the target compounds. The mechanism of inhibition was analyzed by recording substrate concentration—enzymatic reaction rate curves in the presence of different concentrations of compounds **15** and **19**. Analysis confirmed a non-competitive type of inhibition (*p* < 0.05) for both compounds. With increasing concentration of inhibitor, apparent *V*_max_ decreased and *K*_m_ remained unchanged. Figure 3 shows Lineweaver-Burk reciprocal plots of measured data. A *K*_i_ value of 1.331 ± 0.125 μM and 0.4533 ± 0.0251 μM was estimated by the nonlinear regression analysis for **15** and **19**, respectively. Such a pattern of inhibition is also characteristic for donepezil and it may indicate prevailing interactions of the enzyme with PAS [36]. PAS of AChE is associated with the ability to induce Aβ aggregation, thus, compounds interacting with this region may inhibit such a process and could have additional benefit for the treatment of AD [37].

### 2.4. Molecular Modeling Studies

We performed virtual screening analysis of the target molecules against selected enzymes (**9**, **15**, **19** and **22** for both *h*AChE and *h*BChE) used in *in vitro* evaluation in order to shed light on the structural basis determining the binding modes in the active sites of these cholinesterases and to explain the discrepancy in the affinities of these ligands towards ChEs. Docking simulations were carried out using AutoDock Vina 1.1.2 [38]. The crystal structures of *h*AChE complexed with an inhibitor donepezil and *h*BChE bound with tacrine were taken from RCSB Protein Data Bank (PDB ID: 4EY7 and PDB ID: 4BDS, respectively) [39,40]. These structures were chosen because of the similarity between its inhibitors and the ligands under study. The structures of *h*AChE and *h*BChE models were checked by Protein Preparation Wizard (Maestro Version 10.2.011, Schrödinger, Mannheim, Germany) to reveal missing atoms, bond angle and length deviations, improper torsion angles, steric clashes, isolated water clusters, *etc.* which could disturb the molecular docking calculations [41,42]. Structural water molecules were excluded from docking calculations.

**Figure 4:**
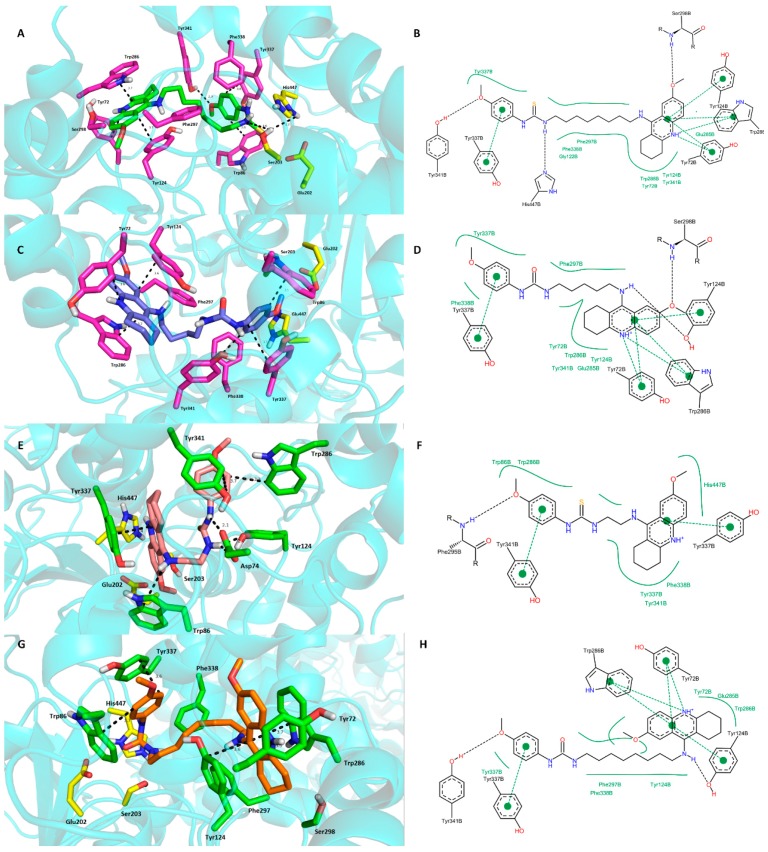
Docking results for the novel 7-MEOTA-*p*-anisidine hybrids (**9**, **15**, **19** and **22**) within *h*AChE active site (PDB ID: 4EY7). (**A**)—Superimposed analogue **15** (green carbon atoms); (**C**)—spatial orientation of **19** (blue carbon atoms); (**E**)—superimposed ligand **9** (salmon pink carbon atoms); (**G**)—superimposed analogue **22** (orange carbon atoms); Generally to (**A**,**C**,**E**,**G**)—important amino acid residues involved in the ligand-enzyme interactions are displayed as purple carbon atoms (**A**,**C**) or as green carbon atoms (**E**,**G**); catalytic triad residues (Glu202, Ser203, His447) are shown in yellow, rest of the enzyme is represented as blue cartoon; (**B**,**D**,**F**,**H**)—2D representation of binding modes of **15**, **19**, **9** and **22**, respectively. Figures (**B**,**D**,**F**,**H**) were created with PoseView software [43]; figure (**A**,**C**,**E**,**G**) were generated with PyMol 1.5.0.4 (The PyMOL Molecular Graphics System, Version 1.5.0.4 Schrödinger, LLC, Mannheim, Germany).

The docking simulations revealed favorable interactions for the highlighted inhibitors involved in the study (**15**, **19**) in the *h*AChE active site with many similarities in their binding modes (Figure 4A,C). The ligands are well-accommodated in the cavity spanning from the bottom through the bottleneck towards the entrance of the enzyme.

Thiourea hybrid **15** revealed dual binding site character inhibition with a distally lodged tetrahydroacridine core in the PAS of the *h*AChE while the *p*-anisidine moiety is oriented towards the CAS region of the enzyme. More in detail, the tetrahydroacridine moiety is sandwiched by π-π interactions between Trp286 (3.7 Å) and Tyr124 (3.7 Å). Charged nitrogen is engaged in cation-π interactions with Tyr72 (3.6 Å). The 7-methoxy appendage further stabilizes ligand anchoring by a weak hydrogen bond to Ser298 (Figure 4B). The tether between the two pharmacophores is delineated mostly by several aromatic residues (Phe297, Tyr341, Phe338) contributing to ligand accommodation by hydrophobic interactions. The thiourea moiety presumably shows a hydrogen bond to catalytic triad residues (Ser203—3.6 Å and His447—3.7 Å) thus enhancing and underlying its importance for ligand-enzyme interaction. At the bottom of the gorge, the phenyl ring of *p*-anisidine revealed favorable parallel π-π (Tyr337—3.6 Å) and T-shaped (Trp86—3.6 Å) interactions. Moreover, the 4-methoxy substituent showed a hydrogen bond to the hydroxyl of Tyr341 (2.9 Å).

**Figure 5:**
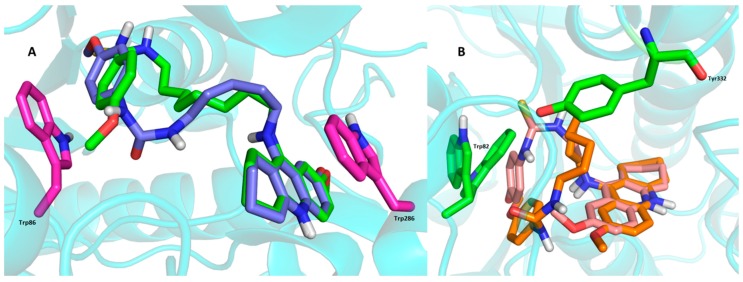
(**A**)—Overlay of the two most populated clusters for **15** (green carbon atoms) and **19** (blue carbon atoms) in the *h*AChE active site. Trp residues representing CAS (Trp86) and PAS (Trp286) of the enzyme are displayed in magenta; (**B**)—Overlay of the two most populated clusters for **9** (salmon pink carbon atoms) and **22** (orange carbon atoms) in the *h*BChE. Trp82 indicates CAS, Tyr332 designates PAS of the *h*BChE. Figure was generated using PyMol 1.5.0.4 (The PyMOL Molecular Graphics System, Version 1.5.0.4 Schrödinger, LLC).

Urea hybrid **19** is bound to the *h*AChE active site in very similar fashion as the **15**-*h*AChE complex. This involves orientation of the 7-methoxytacrine unit into the PAS region with apparent π-π sandwich-like interactions to Trp286 (3.6 Å) and Tyr124 (3.7 Å), and, cation-π binding to Tyr72 (3.6 Å). *p*-Anisidine is located at the bottom of the gorge, being stabilized by parallel π-π interactions with Tyr337 (3.7 Å) and T-shaped bonding to Trp86 (3.5 Å) and Phe338 (3.8 Å). Contrary to the thiourea moiety in the **15**-*h*AChE complex, the urea moiety displayed only hydrogen bond formation to OH from Tyr341 (2.4 Å) with unattached catalytic triad. The shorter chain of **19** plausibly does not permit *p*-anisidine to reach the catalytic triad residues. However, when taking into consideration the very similar data from *in vitro* and calculated affinities by AutoDock Vina (−13.3 kcal/mol and −13.0 kcal/mol for **15** and **19**, respectively), these results suggests that **19** furnished better arrangement with minor restrictions to the enzyme than its longer-chained thiourea counterpart **15** in the active site gorge. The overlap of the highest energy clusters for **15** and **19** complexed to *h*AChE is displayed in Figure 5.

From the docking studies previously reported by us for tacrine-trolox hybrids, 7-MEOTA moiety has been shown to presumably bind to the PAS of *h*AChE [44]. Moreover, tacrine-trolox hybrids also displayed mixed type inhibition patterns assuming the dual binding site character with balanced interactions to both anionic sites. Based on the docking studies for novel 7‑MEOTA-*p*-anisidine hybrids (mainly observed for **19**), we presume that the 7-MEOTA moiety allowed more robust interactions within the PAS region and minor interactions in the CAS (provided by *p*-anisidine) which may also explain the non-competitive behavior obtained from the kinetic analysis with prevailing interactions within the PAS of *h*AChE.

We also investigated the spatial orientation of **9** and **22** trying to explain their rather low potency against *h*AChE. As shown in Figure 4, thiourea hybrid **9** revealed opposite accommodation in the *h*AChE cavity compared to **15** and **19**. The output for ligand **9** reported in Figure 4E,F displayed the 7-MEOTA moiety lodging in the CAS while the *p*-anisidine protrudes out of the gorge. The 7-MEOTA moiety is bound with parallel π-π interactions to Tyr337 (3.4 Å) and in T-shaped orientation to Trp86 (3.7 Å). No interactions with catalytic triad residues can be observed. *p*-Anisidine established π-π interactions with Trp286 (3.3 Å) and Tyr341 (3.7 Å). Interestingly, the thiourea group demonstrated favorable polar contacts to the amino group of Asp74 (2.1 Å and 2.4 Å) and phenolic hydroxyl of Tyr124 (2.0 Å). In general, the low potency of **9** against *h*AChE might result from the inverted ligand topology, inability to fully contact the PAS residues with missing cation-π interactions to Tyr72 and sandwiched-like π-π interactions with Trp286 and Tyr124. Last but not least, the catalytic triad remained intact.

On the contrary, urea derivative **22** (Figure 4G,H) is situated in a similar manner to the most active *h*AChE inhibitors under the study, **15** and **19**. The only disparities that can be observed are the missing hydrogen contact to His447 from the catalytic triad and polar contact between Ser298 with the methoxy group. The latter dissension is based upon 180° rotation of 7-MEOTA moiety in the CAS of the enzyme.

The estimated binding energies for **9** and **22** provided by the AutoDock Vina were −12.2 kcal/mol and −12.6 kcal/mol, respectively, thus lying in the lower range compared to ligands **15** and **19**. These results are also consistent with our observations obtained from *in vitro* studies

**Figure 6:**
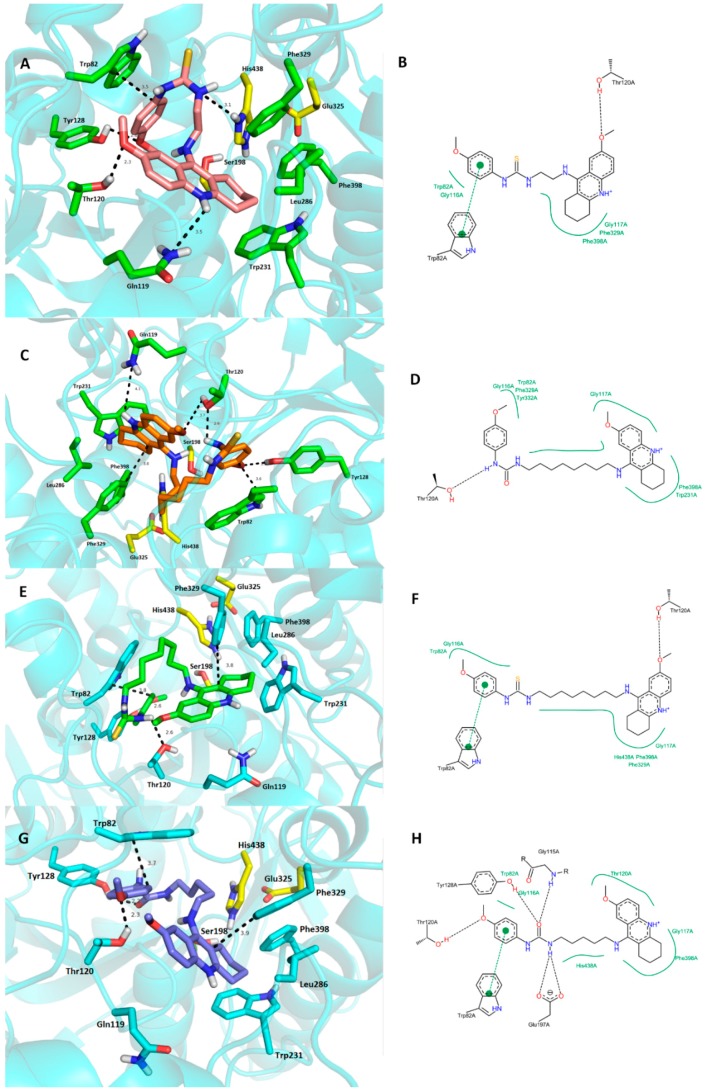
Top scored docking poses for **9**, **22**, **15** and **19** in the *h*BChE (PDB ID: 4BDS) active site. (**A**)—Superimposed analogue **9** (salmon pink carbon atoms); (**C**)—spatial orientation of **22** (orange carbon atoms); (**E**)—superimposed ligand **15** (green carbon atoms); (**G**)—superimposed analogue **19** (dark blue carbon atoms). Generally to (**A**,**C**,**E**,**G**)—important amino acid residues involved in the ligand-enzyme interactions are displayed as green carbon atoms (**A**,**E**) and as light blue carbon atoms (**E**,**G**), catalytic triad residues (Ser198, Glu325, His438) are shown in yellow, rest of the enzyme is represented as blue cartoon; (**B**,**D**,**F**,**H**)—2D representation of binding modes of **9**, **22**, **15** and **19**, respectively. Figures (**B**,**D**,**F**,**H**) were created with PoseView software [43]; figures (**A**,**C**,**E**,**G**) were generated with PyMol 1.5.0.4 (The PyMOL Molecular Graphics System, Version 1.5.0.4 Schrödinger, LLC).

Examination of the complex structures revealed the molecular basis of the high affinity binding of **9** and **22** to *h*BChE (PDB ID: 4BDS) active site. These were selected based upon their *in vitro* IC_50_ values (Figure 6) [39].

Thiourea analogue **9** resides deep in the gorge of the *h*BChE with major arene-to-arene (Trp82—3.5 Å, Phe329—4.2 Å) and hydrogen bond intearctions between the *p*-anisidine methoxy group to OH from Tyr128 (2.6 Å) of the catalytic anionic site residues. The 7-MEOTA moiety of **9** protrudes out of the gorge, while the *p*-anisidine is oriented proximally to bottom of the gorge. Due to the short tether composed of two methylenes, ligand **9** does not provide any interaction at the cavity entrance leaving the PAS residues (Asp70 and Tyr332) unaffected. The thhiourea moiety also contributes to ligand-enzyme stability by hydrogen-bond formation to His438 (3.1 Å). Other catalytic triad residues (Ser198, Glu325) are not involved in the ligand anchoring.

Very close ligand binding can be seen for urea hybrid **22**. Docking simulation placed the ligand in almost identical topology compared to **9** with a distorted linkage between the *p*-anisidine and 7-MEOTA moieties. This allowed contact with the PAS region by weak hydrophobic interactions to Tyr332 (4.2 Å) and Asp70 (4.3 Å). Very similarly, ligand **22** occupies the proximity of CAS residues where it parallelly-stacks to Trp82 and Phe329 (3.6 Å and 3.8 Å, respectively, π-π interaction) and forms hydrogen bonding between OH from Tyr128 and the methoxy group of *p*-anisidine (2.6 Å). In this case, catalytic triad residues do not play a pivotal role in ligand-enzyme constriction. Thr120 seems to be play a very important role which stabilizes the distorted ligand placement by forming hydrogen bonds to both the methoxy group of the tetrahydroacridine unit (3.7 Å) and the urea group (2.6 Å). Like the general *h*BChE docking studies, estimated binding energies by AutoDock Vina software were −10.1 kcal/mol and −10.2 kcal/mol for **9** and **22**, respectively, which is in accordance with the very close IC_50_ values obtained from *in vitro* studies. The overlapped structures of both ligands under survey are displayed in Figure 5B.

We also docked ligands **15** and **19** into the *h*BChE active site in order to clarify their low affinity towards this enzyme with respect to the highlighted *h*BChE inhibitors in this study, *i.e.*, derivatives **9** and **22**. In all cases, the 7-MEOTA moiety accommodated very close spatial orientation near Phe329. The disparity in the bindings of all ligands results from the chain alignment and imposition of the *p*-anisidine moiety. However, as depicted in Figure 6E–H no clear diversity trends in the enzyme-ligand interactions can be seen when compared to the **9**- and **22**-*h*BChE complexes, so we assume that this cannot be explained by the simplistic method exploited by molecular modeling studies. A more valuable approach to elucidate this problem is through molecular dynamics to include the influence of the temperature and water in the molecular system which is beyond the scope of this study.

## 3. Experimental Section

### 3.1. General Chemistry

All the chemical reagents used were purchased from Sigma-Aldrich (Prague, Czech Republic). Solvents for synthesis were obtained from Penta Chemicals Co. (Prague, Czech Republic). The course of the reactions was monitored by thin layer chromatography (TLC) on aluminium plates precoated with silica gel 60 F254 (Merck, Prague, Czech Republic) and then visualized by UV 254. Melting points were determined on a melting point apparatus M-565 (Büchi, Flawil, Switzerland) and are uncorrected. NMR spectra of target compounds were recorded on Varian Mercury VX BB 300 (operating at 300 MHz for ^1^H and 75 MHz for ^13^C) or on Varian S500 spectrometer (operating at 500 MHz for ^1^H and 126 MHz for ^13^C; Varian Co. Palo Alto, CA, USA). Chemical shifts are reported in parts per million (ppm). Spin multiplicities are given as s (singlet), bs (broad singlet), d (doublet), dd (doublet of doublets), t (triplet), q (quartet), or m (multiplet). The coupling constants (*J*) are reported in Hertz (Hz). High-resolution mass spectra (HRMS) were determined by an Q Exactive Plus hybrid quadrupole-orbitrap spectrometer (Thermo Fisher Scientific, Waltham, MA USA).

#### 3.1.1. General Synthetic Procedure for 7-MEOTA-*p*-anisidine Thiourea 2,3-Dihydroxysuccinate Hybrids **9**–**15**

*N*-(7-methoxy-1,2,3,4-tetrahydroacridin-9-yl)alkane-1,ω-diamines **2**–**8** (10 mmol) and 1-isothio-cyanato-4-methoxybenzene (**1**, 12 mmol) were dissolved in chloroform and stirred 24 h at room temperature. The crude reaction mixture was evaporated to dryness and purified via column chromatography (9:1 chloroform/methanol as eluent). Pure bases were converted into tartrate salts by addition of equimolar l-(+)-tartaric acid and further stirring in absolute ethanol (10 mL) for 24 h. 7-MEOTA-*p*-anisidine thiourea 2,3‑dihydroxysuccinates **9**–**15** were thus obtained as white-yellow solids in low-to-moderate yields (15%–42%).

*3-{2-[(7-Methoxy-1,2,3,4-tetrahydroacridin-9-yl)amino]ethyl}-1-(4-methoxyphenyl)thiourea-2,3‑dihydroxy-succinate* (**9**). Yield: 32%; m.p. = 200.3–201.8 °C; ^1^H-NMR (300 MHz, DMSO-*d*_6_): δ (ppm) 8.11 (bs, 1H), 7.79 (d, *J* = 9.9 Hz, 1H), 7.23–7.15 (m, 2H), 7.06–6.96 (m, 2H), 6.85–6.75 (m, 2H), 6.41 (bs, 1H), 4.46 (bs, 1H), 4.09 (s, 2H), 4.03–3.92 (m, 2H), 3.88 (s, 3H), 3.75 (s, 3H), 3.67–3.55 (m, 2H), 2.97 (t, *J* = 5.7 Hz, 2H), 2.67 (t, *J* = 5.8 Hz, 2H), 1.91–1.73 (m, 4H); ^13^C-NMR (75 MHz, DMSO-*d*_6_): δ (ppm) 180.11, 174.12, 158.82, 156.32, 155.91, 149.54, 131.45, 129.64, 128.33, 127.41, 120.91, 120.70, 117.54, 115.09, 101.10, 71.57, 55.73, 55.44, 48.53, 45.68, 33.24, 25.50, 22.94, 22.59; HRMS [M + H]^+^: 437.1969 (calculated for [C_24_H_29_N_4_O_2_S]^+^: 437.1967).

*3-{3-[(7-Methoxy-1,2,3,4-tetrahydroacridin-9-yl)amino]propyl}-1-(4-methoxyphenyl)thiourea-2,3‑dihydroxy-succinate* (**10**). Yield: 22%; m.p. = 112.4–114.5 °C; ^1^H-NMR (300 MHz, DMSO-*d*_6_): δ (ppm) 8.01 (bs, 1H), 7.85 (d, *J* = 9.9 Hz, 1H), 7.25–7.16 (m, 2H), 7.10–7.01 (m, 2H), 6.70–6.59 (m, 2H), 4.66 (bs, 1H), 4.10 (s, 2H), 3.89 (s, 3H), 3.88–3.82 (m, 2H), 3.60 (s, 3H), 3.54–3.42 (m, 2H), 3.07–2.96 (m, 2H), 2.74–2.60 (m, 2H), 1.94–1.78 (m, 6H); ^13^C-NMR (75 MHz, DMSO-*d*_6_): δ (ppm) 181.78, 173.76, 158.85, 156.33, 156.29, 128.13, 128.05, 121.13, 120.93, 120.86, 114.81, 100.98, 71.24, 55.71, 55.24, 45.69, 43.85, 30.29, 29.65, 25.08, 22.91, 22.59; HRMS [M + H]^+^: 451.2138 (calculated for [C_25_H_31_N_4_O_2_S]^+^: 451.2123).

*3-{4-[(7-Methoxy-1,2,3,4-tetrahydroacridin-9-yl)amino]butyl}-1-(4-methoxyphenyl)thiourea-2,3‑dihydroxy-succinate* (**11**). Yield: 15%; m.p. = 109.7–111.9 °C; ^1^H-NMR (300 MHz, DMSO-*d*_6_): δ (ppm) 7.94 (bs, 1H), 7.83 (d, *J* = 9.9 Hz, 1H), 7.23–7.18 (m, 2H), 7.14–7.08 (m, 2H), 6.87–6.81 (m, 2H), 6.30 (bs, 1H), 4.07 (s, 2H), 3.89 (s, 3H), 3.77 (s, 3H), 3.69–3.62 (m, 2H), 3.48 (t, *J* = 6.4 Hz, 2H), 3.04–2.98 (m, 2H), 2.71–2.63 (m, 2H), 1.93–1.82 (m, 4H), 1.75–1.62 (m, 4H).; ^13^C-NMR (125 MHz, DMSO-*d*_6_): δ (ppm) 181.53, 174.24, 158.67, 156.15, 150.40, 150.37, 128.59, 127.53, 127.49, 120.92, 120.61, 116.54, 115.02, 114.98, 101.71, 71.59, 55.64, 55.47, 48.10, 44.64, 32.70, 28.63, 26.57, 24.80, 22.84, 22.40; HRMS [M + H]^+^: 465.2266 (calculated for [C_26_H_33_N_4_O_2_S]^+^: 465.2280).

*3-{5-[(7-Methoxy-1,2,3,4-tetrahydroacridin-9-yl)amino]pentyl}-1-(4-methoxyphenyl)thiourea-2,3‑dihydroxy-succinate* (**12**). Yield: 42%; m.p. = 127.1–129.5 °C; ^1^H-NMR (300 MHz, DMSO-*d*_6_): δ (ppm) 9.63 (bs, 1H), 7.90 (bs, 1H), 7.76 (d, *J* = 9.9 Hz, 1H), 7.56 (d, *J* = 2.6 Hz, 1H), 7.37 (dd, *J* = 9.9, 2.6 Hz, 1H), 7.27–7.16 (m, 2H), 6.92–6.78 (m, 2H), 4.03 (s, 2H), 3.88 (s, 3H), 3.71 (s, 3H), 3.61 (t, *J* = 6.5 Hz, 2H), 3.47–3.35 (m, 2H), 2.99–2.86 (m, 2H), 2.78–2.63 (m, 2H), 1.88–1.72 (m, 4H), 1.72–1.59 (m, 2H), 1.59–1.43 (m, 2H), 1.41–1.25 (m, 2H).; ^13^C-NMR (75 MHz, DMSO-*d*_6_): δ (ppm) 180.54, 174.39, 156.13, 155.95, 152.69, 151.91, 136.50, 132.09, 125.52, 124.39, 122.24, 118.68, 113.63, 113.40, 102.64, 71.81, 55.63, 55.13, 48.54, 46.98, 30.12, 29.83, 28.22, 24.81, 23.58, 22.06, 21.11; HRMS [M + H]^+^: 479.2416 (calculated for [C_27_H_35_N_4_O_2_S]^+^: 479.2436).

*3-{6-[(7-Methoxy-1,2,3,4-tetrahydroacridin-9-yl)amino]hexyl}-1-(4-methoxyphenyl)thiourea-2,3‑dihydroxy-succinate* (**13**). Yield: 21%; m.p. = 177.0–178.3 °C; ^1^H-NMR (500 MHz, DMSO-*d*_6_): δ (ppm) 9.53 (bs, 1H), 7.79 (bs, 1H), 7.73 (d, *J* = 9.8 Hz, 1H), 7.53 (d, *J* = 2.7 Hz, 1H), 7.34 (dd, *J* = 9.8, 2.7 Hz, 1H), 7.27–7.18 (m, 2H), 6.91–6.81 (m, 2H), 6.42 (bs, 1H), 3.99 (s, 2H), 3.88 (s, 3H), 3.71 (s, 3H), 3.55 (t, *J* = 6.7 Hz, 2H), 3.46–3.31 (m, 2H), 2.92 (t, *J* = 5.8 Hz, 2H), 2.70 (t, *J* = 5.7 Hz, 2H), 1.86–1.72 (m, 4H), 1.69–1.55 (m, 2H), 1.55–1.41 (m, 2H), 1.40–1.21 (m, 4H); ^13^C-NMR (125 MHz, DMSO-*d*_6_): δ (ppm) 180.52, 174.34, 156.14, 155.80, 152.57, 152.02, 137.83, 132.13, 125.38, 121.72, 119.13, 114.05, 113.63, 102.41, 71.65, 55.53, 55.10, 47.04, 43.59, 30.43, 30.37, 28.43, 26.02, 25.92, 24.84, 22.15, 21.35; HRMS [M + H]^+^: 493.2596 (calculated for [C_28_H_37_N_4_O_2_S]^+^: 493.2593).

*3-{7-[(7-Methoxy-1,2,3,4-tetrahydroacridin-9-yl)amino]heptyl}-1-(4-methoxyphenyl)thiourea-2,3‑dihydroxy-succinate* (**14**). Yield: 33%; m.p. = 98.1–100.3 °C; ^1^H-NMR (500 MHz, DMSO-*d*_6_): δ (ppm) 7.97 (bs, 1H), 7.87 (d, *J* = 9.9 Hz, 1H), 7.25 (d, *J* = 2.7 Hz, 1H), 7.21 (dd, *J* = 9.9, 2.7 Hz, 1H), 7.16–7.10 (m, 2H), 6.90–6.84 (m, 2H), 6.10 (bs, 1H), 4.21 (bs, 1H), 4.08 (s, 2H), 3.89 (s, 3H), 3.77 (s, 3H), 3.63–3.52 (m, 2H), 3.46 (t, *J* = 6.6 Hz, 2H), 3.09–2.96 (m, 2H), 2.76–2.62 (m, 2H), 1.94–1.80 (m, 4H), 1.72–1.58 (m, 2H), 1.57–1.46 (m, 2H), 1.44–1.19 (m, 6H); ^13^C-NMR (125 MHz, DMSO-*d*_6_): δ (ppm) 181.26, 174.25, 158.59, 156.00, 154.92, 150.77, 128.88, 128.51, 127.5, 120.84, 120.48, 116.10, 116.08, 114.970, 102.021, 72.35, 55.53), 55.45, 48.77, 45.11, 32.68, 31.46, 28.83, 28.76, 26.66, 26.51, 24.59, 22.81, 22.34; HRMS [M + H]^+^: 507.2766 (calculated for [C_29_H_39_N_4_O_2_S]^+^: 507.2749).

*3-{8-[(7-Methoxy-1,2,3,4-tetrahydroacridin-9-yl)amino]octyl}-1-(4-methoxyphenyl)thiourea-2,3‑dihydroxy-succinate* (**15**). Yield: 16%; m.p. = 89.3–91.9 °C; ^1^H-NMR (300 MHz, DMSO-*d*_6_): δ (ppm) 8.07 (bs, 1H), 7.95 (d, *J* = 9.8 Hz, 1H), 7.31 (d, *J* = 2.7 Hz, 1H), 7.22 (dd, *J* = 9.8, 2.7 Hz, 1H), 7.18–7.10 (m, 2H), 6.91–6.81 (m, 2H), 6.23 (bs, 1H), 4.10 (S, 2H), 3.89 (s, 3H), 3.76 (s, 3H), 3.62–3.48 (m, 4H), 3.11–3.00 (m, 2H), 2.73–2.63 (m, 2H), 1.94–1.80 (m, 4H), 1.76–1.61 (m, 2H), 1.57–1.44 (m, 2H), 1.43–1.17 (m, 8H).; ^13^C-NMR (75 MHz, DMSO-*d*_6_): δ (ppm) 181.28, 174.49, 158.44, 156.15, 153.79, 151.65, 131.42, 129.17, 127.41, 127.07, 121.42, 119.81, 114.98, 114.85, 102.37, 71.76, 55.62, 55.44, 48.63, 45.10, 40.92, 31.74, 31.43, 28.95, 28.86, 26.61, 26.48, 24.50, 22.63, 22.01; HRMS [M + H]^+^: 521.2935 (calculated for [C_30_H_41_N_4_O_2_S]^+^: 521.2906).

#### 3.1.2. General Synthetic Procedure for 7-MEOTA-*p*-anisidine Urea 2,3-Dihydroxysuccinate Hybrids **16**–**22**

7-MEOTA-*p*-anisidine thioureas (**9**–**15**, free bases, 10 mmol,) were treated with 2,4,6‑trimethyl-benzonitrile-*N*-oxide (11 mmol) in dichloromethane (10 mL) at room temperature for 24 h. The solvent was evaporated and crude residue was purified with column chromatography using chloroform/methanol (9:1) as eluent. Resulting intermediates were converted into the title compounds by treating with equimolar l-(+)-tartaric acid in absolute ethanol for 24 h at room temperature. This led to the formation of 7-MEOTA-*p*-anisidine urea 2,3-dihydroxysuccinate hybrids **16**–**22** as white-to-yellow powders in low-to-moderate yields (13%–46%)

*3-{2-[(7-Methoxy-1,2,3,4-tetrahydroacridin-9-yl)amino]ethyl}-1-(4-methoxyphenyl)urea-2,3‑dihydroxy-succinate* (**16**). Yield: 27%; m.p. = 198.1–200.5 °C; ^1^H-NMR (500 MHz, DMSO-*d*_6_): δ (ppm) 8.74 (bs, 1H), 7.75 (m, 1H), 7.59 (m, 1H), 7.36 (m, 1H), 7.26 (m, 2H), 6.80 (m, 2H), 4.02 (s, 2H), 3.87 (s, 3H), 3.79–3.70 (m, 2H), 3.69 (s, 3H), 3.48–3.34 (m, 2H), 3.00–2.85 (m, 2H), 2.82–2.69 (s, 2H), 1.87–1.61 (m, 4H); ^13^C-NMR (125 MHz, DMSO-*d*_6_): δ (ppm) 174.14, 156.53, 155.94, 153.98, 152.68, 152.12, 136.77, 133.24, 124.57, 122.16, 119.62, 118.75, 113.71, 113.45, 102.40, 71.61, 55.54, 55.04, 48.83, 30.05, 24.80, 22.09, 21.12; HRMS [M + H]^+^: 421.2192 (calculated for [C_24_H_29_N_4_O_3_]^+^: 421.2195).

*3-{3-[(7-Methoxy-1,2,3,4-tetrahydroacridin-9-yl)amino]propyl}-1-(4-methoxyphenyl)urea-2,3‑dihydroxy-succinate* (**17**). Yield: 13%; m.p. = 67.5–69.3 °C; ^1^H-NMR (500 MHz, DMSO-*d*_6_): δ (ppm) 7.87 (bs, 1H), 7.79 (d, *J* = 9.8 Hz, 1H), 7.38 (d, *J* = 2.7 Hz, 1H), 7.25–7.21 (m, 2H), 7.18 (dd, *J* = 9.8, 2.7 Hz, 1H), 6.76–6.69 (m, 2H), 6.09 (bs, 1H), 5.51 (bs, 1H), 4.05 (s, 2H), 3.86 (s, 3H), 3.71 (s, 3H), 3.53–3.45 (m, 2H), 3.41–3.33 (m, 2H), 3.00–2.92 (m, 2H), 2.72–2.63 (m, 2H), 1.86–1.76 (m, 4H), 1.75–1.67 (m, 2H).; ^13^C-NMR (125 MHz, DMSO-*d*_6_): δ (ppm) 174.44, 157.66, 156.39, 155.91, 154.53, 151.24, 140.46, 131.99, 127.36, 122.71, 121.47, 120.49, 115.81, 114.17, 101.65, 71.68, 55.66, 55.43, 44.58, 36.89, 32.01, 29.67, 25.22, 22.83, 22.23; HRMS [M + H]^+^: 435.2368 (calculated for [C_25_H_31_N_4_O_3_]^+^: 435.2351).

*3-{4-[(7-Methoxy-1,2,3,4-tetrahydroacridin-9-yl)amino]butyl}-1-(4-methoxyphenyl)urea*-*2,3‑dihydroxy-succinate* (**18**). Yield: 25%; m.p. = 99.9–101.2 °C; ^1^H-NMR (500 MHz, DMSO-*d*_6_): δ (ppm) 7.83 (d, *J* = 9.9 Hz, 1H), 7.59 (bs, 1H), 7.29 (d, *J* = 2.6 Hz, 1H), 7.25–7.21 (m, 2H), 7.16 (dd, *J* = 9.9, 2.6 Hz, 1H), 6.76–6.72 (m, 2H), 5.84 (bs, 1H), 4.09 (s, 2H), 3.86 (s, 3H), 3.72 (s, 3H), 3.50 (t, *J* = 6.6 Hz, 2H), 3.28–3.20 (m, 2H), 3.03–2.93 (m, 2H), 2.67–2.55 (m, 2H), 1.87–1.74 (m, 4H), 1.74–1.64 (m, 2H), 1.62–1.50 (m, 2H); ^13^C-NMR (125 MHz, DMSO-*d*_6_): δ (ppm) 174.21, 156.92, 156.31, 155.78, 153.26, 151.82, 132.14, 122.47, 122.46, 121.63, 121.62, 119.52, 119.49, 114.21, 102.36, 71.67, 55.73, 55.46, 47.96, 39.38, 29.68, 28.61, 27.52, 24.65, 22.57, 21.84; HRMS [M + H]^+^: 449.2517 (calculated for [C_26_H_33_N_4_O_3_]^+^: 449.2508).

*3-{5-[(7-Methoxy-1,2,3,4-tetrahydroacridin-9-yl)amino]pentyl}-1-(4-methoxyphenyl)urea-2,3‑dihydroxy-succinate* (**19**). Yield: 37%; m.p. = 90.3–92.8 °C; ^1^H-NMR (300 MHz, DMSO-*d*_6_): δ (ppm) 7.88 (d, *J* = 9.8 Hz, 1H), 7.47 (bs, 1H), 7.30 (d, *J* = 2.6 Hz, 1H), 7.25–7.16 (m, 3H), 6.78–6.69 (m, 2H), 5.59 (bs, 1H), 4.68 (bs, 1H), 4.05 (s, 2H), 3.87 (s, 3H), 3.71 (s, 3H), 3.56–3.44 (m, 2H), 3.26–3.15 (m, 2H), 3.04–2.94 (m, 2H), 2.69–2.60 (m, 2H), 1.88–1.77 (m, 4H), 1.74–1.60 (m, 2H), 1.55–1.35 (m, 4H); ^13^C-NMR (75 MHz, DMSO-*d*_6_): δ (ppm) 174.25, 156.91, 156.23, 155.94, 153.92, 151.73, 132.04, 131.47, 127.10, 122.85, 121.49, 119.93, 115.26, 114.24, 102.44, 71.52, 55.66, 55.45, 48.46, 39.57, 31.81, 31.01, 29.74, 24.58, 24.02, 22.63, 22.02; HRMS [M + H]^+^: 463.2677 (calculated for [C_27_H_35_N_4_O_3_]^+^: 463.2664).

*3-{6-[(7-Methoxy-1,2,3,4-tetrahydroacridin-9-yl)amino]hexyl}-1-(4-methoxyphenyl)urea-2,3‑dihydroxy-succinate* (**20**). Yield: 46%; m.p. = 100.9–102.5 °C; ^1^H-NMR (500 MHz, DMSO-*d*_6_): δ (ppm) 7.82 (d, *J* = 9.8 Hz, 1H), 7.36 (bs, 1H), 7.26–7.25 (m, 1H), 7.23–7.19 (m, 3H), 6.80–6.72 (m, 2H), 5.37 (bs, 1H), 4.04 (s, 2H), 3.87 (s, 3H), 3.72 (s, 3H), 3.41 (t, *J* = 6.6 Hz, 2H), 3.21–3.11 (m, 2H), 3.03–2.94 (m, 2H), 2.71–2.64 (m, 2H), 1.92–1.81 (m, 4H), 1.66–1.54 (m, 2H), 1.46–1.39 (m, 2H), 1.39–1.22 (m, 8H).; ^13^C-NMR (125 MHz, DMSO-*d*_6_): δ (ppm) 174.29, 156.81, 156.02, 155.99, 155.27, 150.70, 141.91, 131.97, 128.71, 122.98, 120.81, 120.68, 116.29, 114.27, 102.05, 71.53, 55.55, 55.44, 48.59, 39.76, 32.89, 31.49, 30.08, 26.41, 26.37, 24.69, 22.88, 22.49; HRMS [M + H]^+^: 477.2844 (calculated for [C_28_H_37_N_4_O_3_]^+^: 477.2821).

*3-{7-[(7-Methoxy-1,2,3,4-tetrahydroacridin-9-yl)amino]heptyl}-1-(4-methoxyphenyl)urea-2,3‑dihydroxy-succinate* (**21**). Yield: 23%; m.p. = 97.3–99.6 °C; ^1^H-NMR (300 MHz, DMSO-*d*_6_): δ (ppm) 7.87 (d, *J* = 9.8 Hz, 1H), 7.75 (bs, 1H), 7.32 (d, *J* = 2.7 Hz, 1H), 7.26–7.18 (m, 3H), 6.76–6.69 (m, 2H), 4.02 (s, 2H), 3.88 (s, 3H), 3.70 (s, 3H), 3.52 (t, *J* = 6.7 Hz, 2H), 3.22–3.07 (m, 2H), 3.06–2.94 (m, 2H), 2.73–2.60 (m, 2H), 1.92–1.76 (m, 4H), 1.72–1.57 (m, 2H), 1.47–1.15 (m, 8H); ^13^C-NMR (75 MHz, DMSO-*d*_6_): δ (ppm) 174.39, 156.91, 156.22, 155.56, 153.78, 151.93, 132.41, 131.44, 126.83, 122.29, 121.59, 119.84, 114.97, 114.10, 102.43, 71.57, 55.64, 55.42, 48.39, 39.83, 31.65, 31.22, 29.86, 29.65, 28.58, 26.41, 24.56, 22.63, 22.00; HRMS [M + H]^+^: 477.2844 (calculated for [C_28_H_37_N_4_O_3_]^+^: 477.2821).

*3-{8-[(7-Methoxy-1,2,3,4-tetrahydroacridin-9-yl)amino]octyl}-1-(4-methoxyphenyl)urea-2,3‑dihydroxy-succinate* (**22**). Yield: 39%; m.p. = 83.0–85.9 °C; ^1^H-NMR (300 MHz, DMSO-*d*_6_): δ (ppm) 7.92–7.84 (m, 2H), 7.34 (d, *J* = 2.7 Hz, 1H), 7.26–7.18 (m, 3H), 6.75–6.66 (m, 2H), 5.78 (bs, 1H),4.05 (s, 2H), 3.88 (s, 3H), 3.69 (s, 3H), 3.60–3.51 (m, 2H), 3.17–3.08 (m, 2H), 3.04–2.96 (m, 2H), 2.71–2.62 (m, 2H), 1.89–1.78 (m, 4H), 1.71–1.57 (m, 2H), 1.43–1.13 (m, 10H); ^13^C-NMR (75 MHz, DMSO-*d*_6_): δ (ppm) 174.28, 156.96, 156.22, 155.40, 153.40, 152.19, 139.27, 132.50, 126.35, 122.05, 121.73, 119.62, 114.63, 114.02, 102.52, 71.57, 55.65, 55.39, 48.38, 39.84, 31.36, 29.96, 29.64, 28.83, 28.80, 26.43, 24.54, 22.64, 22.58, 21.91; HRMS [M + H]^+^: 491.2972 (calculated for [C_29_H_39_N_4_O_3_]^+^: 491.2977).

### 3.2. Biochemical Studies

#### 3.2.1. *In Vitro* Anti-Cholinesterase Assay

The AChE and BChE inhibitory activity of the tested compounds was determined using a modified Ellman method [32]. Human recombinant acetylcholinesterase (*h*AChE; EC 3.1.1.7, human plasma butyrylcholinesterase (*h*BChE; EC 3.1.1.8), 5,5′-dithiobis(2-nitrobenzoic acid) (Ellman’s reagent, DTNB), phosphate buffer (PB, pH 7.4), acetylthiocholine (ATCh), and butyrylthiocholine (BTCh), were purchased from Sigma-Aldrich (Prague, Czech Republic). For measuring purposes–polystyrene Nunc 96-well microplates with flat bottom shape (Thermo Fisher Scientific, Waltham, MA USA) were utilized. All the assays were carried out in 0.1 M KH_2_PO_4_/K_2_HPO_4_ buffer, pH 7.4. Enzyme solutions were prepared at activity 2.0 units/mL in 2 mL aliquots. The assay medium (100 μL) consisted of 40 μL of 0.1 M phosphate buffer (pH 7.4), 20 μL of 0.01 M DTNB, 10 μL of enzyme, and 20 μL of 0.01 M substrate (ATCh iodide solution). Assay solutions with inhibitor (10 μL, 10^−3^–10^−9^ M) were preincubated for 5 min. The reaction was started by addition of 20 μL of substrate (ATCh for *h*AChE, BTCh for *h*BChE). The enzyme activity was determined by measuring the increase in absorbance at 412 nm at 37 °C at 2 min intervals—using a multi-mode Synergy 2 microplate reader (Bio-Tek, Winooski, VT, USA). Each concentration was assayed in triplicate. The obtained data were used to compute percentage of inhibition (I; Equation (1)):

(1) $$I=\left(1-\frac{\Delta A_{i}}{\Delta A_{0}}\right)\times 100\text{ [\%]}$$

Δ*A*_i_ indicates absorbance change provided by cholinesterase exposed to AChE inhibitors and Δ*A*_0_ indicates absorbance change caused by intact cholinesterase (phosphate buffer was used instead of AChE inhibitor solution). Inhibition potency of tested compounds was expressed as IC_50_ value (concentration of inhibitor, which causes 50% cholinesterase inhibition). Calculations were performed using the Microsoft Excel software (Microsoft Inc., Redmond, WA, USA) and GraphPad Prism version 5.02 for Windows (GraphPad Software, San Diego, CA, USA; www.graphpad.com).

#### 3.2.2. Kinetic Study of AChE Inhibition

The kinetic study of AChE inhibition was performed by using Ellman’s method (described above) [32]. The type of inhibition was elucidated from the nonlinear regression analysis. Results for each type model of inhibition (competitive, noncompetitive, uncompetitive and mixed) were compared with sum-of-squares *F*-test. For the measurements, following concentrations of substrate were used: 78.13, 156.3, 312.5 and 625 μM. *V*_max_ and *K*_m_ values, respectively, of the Michaelis-Menten kinetics and *K*_i_ were calculated by non-linear regression from the substrate velocity curves. Linear regression was used for calculation of Lineweaver-Burk plots. All calculations were performed using the GraphPad Prism software.

### 3.3. Molecular Modeling Studies

From the online PDB database (www.pdb.org) models of *h*AChE (PDB ID: 4EY7, resolution: 2.35 Å) and *h*BChE (PDB ID: 4BDS, resolution: 2.10 Å) were downloaded and prepared for flexible molecular docking by MGL Tools utilities. The preparation of this receptor involved removal of the surplus copies of the enzyme chains, non-bonded inhibitors, addition of polar hydrogens and merging of non-polar ones. Default Gasteiger charges were assigned to all atoms. Flexible parts of the enzymes were determined by a spherical selection of residues (R = 11 Å) approximately around the center of the active site. In the same points the centers of the grid box of 33 × 33 × 33 Å were positioned. The rotatable bonds in the flexible residues were detected automatically by AutoDock Tools 1.5.4 program (The Scripps Research Institute, La Jolla, CA, USA). Given the limitation of the program used for flexible molecular docking, water molecules had to be removed from the system. The flexible receptor parts contained 40 residues for *h*AChE and 39 residues for *h*BChE. Following xyz coordinates of the grid box centers were applied: *h*AChE (10.698, −58.115, −23.192); *h*BChE (140.117, 122.247, 38.986). The studied ligands were firstly drawn in HyperChem 8.0, then manually protonated as suggested by MarvinSketch 6.2.0. software (http://www.chemaxon.com, ChemAxon, Budapest, Hungary), geometrically optimized by semi-empirical quantum-chemistry PM3 method and stored as pdb files. The structures of the ligands were processed for docking in a similar way as abovementioned flexible parts of the receptor by AutoDock Tools 1.5.4 program (The Scripps Research Institute, La Jolla, CA, USA). Molecular docking was carried out in AutoDock Vina 1.1.2 program utilizing computer resources of the Czech National Grid Infrastructure MetaCentrum (Prague, Czech Republic). The search algorithm of AutoDock Vina efficiently combines a Markov chain Monte Carlo like method for the global search and a Broyden-Fletcher-Goldfarb-Shano gradient approach for the local search [38]. It is a type of memetic algorithm based on interleaving stochastic and deterministic calculations [45]. Each docking task was repeated 30 times with the exhaustiveness parameter set to 16, employing 16 CPU in parallel multithreading. From the obtained results, the solutions reaching the minimum predicted Gibbs binding energy were taken as the top-scoring modes. The graphic representations of the docked poses were rendered in PyMOL 1.5.0.4 (The PyMOL Molecular Graphics System, Version 1.5.0.4 Schrödinger, LLC), 2D diagrams were generated using PoseView software [43].

## 4. Conclusions

While several novel approaches are still awaiting their market launch, AChEIs continue to play an important role in AD therapy and thus represent a major focus of drug development in this field [46]. In this work we followed the rational design of multi-target-directed ligands (MTDLs) approach based upon the fact that multiple interactions among different biological systems may be purposely responsible for the onset and/or progression of the disease [47,48]. To date, no biological data gives supportive evidence of which is the best strategy to follow the intertwined pathological pathways of patient’s brains suffering from AD. Thus, we pursued the MTDLs strategy and report preliminary data for a novel series of 7-MEOTA-*p*-ansidine hybrids. These novel hybrids displayed mostly a non-selective, moderate profile in inhibiting cholinesterases with a non-competitive pattern of inhibition towards *h*AChE. In line with these results, *in silico* studies confirmed the dual binding site character of the selected ligands, with prevailing interactions with the PAS region of *h*AChE. Such a peculiarity might be beneficial in inhibiting the well-known non-cholinergic role of *h*AChE in promotion of Aβ aggregation [37]. However, further tests are needed to fully assess the real potential of the novel 7-MEOTA-*p*-anisidine hybrids. The effect of hybridization in both parts (*i.e.*, tacrine and *p*-anisidine regions) of these hybrids on biological activity will be also established.
